# Prevalence of and Associated Factors for Adult Attention Deficit Hyperactivity Disorder in Young Swiss Men

**DOI:** 10.1371/journal.pone.0089298

**Published:** 2014-02-20

**Authors:** Natalia Estévez, Dominique Eich-Höchli, Michelle Dey, Gerhard Gmel, Joseph Studer, Meichun Mohler-Kuo

**Affiliations:** 1 Institute of Social and Preventive Medicine, Zurich, Switzerland; 2 Department of Neuroradiology, University Hospital Zurich, Zurich, Switzerland; 3 Psychiatric University Hospital Zurich, Zurich, Switzerland; 4 Alcohol Treatment Centre, Lausanne University Hospital CHUV, Lausanne, Switzerland; Alexander Fleming Biomedical Sciences Research Center, Greece

## Abstract

**Objective:**

The present study aimed to measure the prevalence of adult attention deficit hyperactivity disorder (ADHD) in a large, representative sample of young Swiss men and to assess factors associated with this disorder.

**Methods:**

Our sample consisted of 5656 Swiss men (mean age 20 years) who participated in the Cohort Study on Substance Use Risk Factors (C-SURF). ADHD was assessed with the World Health Organization (WHO) adult ADHD Self Report Screener (ASRS). Logistic regression analyses were conducted to assess the association between ADHD and several socio-demographic, clinical and familial factors.

**Results:**

The prevalence of ADHD was 4.0%, being higher in older and French-speaking conscripts. A higher prevalence also was identified among men whose mothers had completed primary or high school/university and those with a family history of alcohol or psychiatric problems. Additionally, adults with ADHD demonstrated impairment in their professional life, as well as considerable mental health impairment.

**Conclusion:**

Our results demonstrate that ADHD is common among young Swiss men. The impairments in function and mental health we observed highlight the need for further support and interventions to reduce burden in affected individuals. Interventions that incorporate the whole family also seem crucial.

## Introduction

According to the DSM-IV-TR [1], Attention Deficit Hyperactivity Disorder (ADHD) is characterized by inattention and/or hyperactivity-impulsivity symptoms, which affect functioning considerably in several life domains. In contrast to an earlier assumption that ADHD only occurs during childhood and adolescence, more recent research has demonstrated that ADHD often persists into adulthood [2], [3]. Adult ADHD is associated with functional impairment relating to professional achievements (e.g., lower levels of education; poorer socioeconomic outcomes) [3]–[9] as well as with more difficulties in relationships (e.g., higher rates of unsuccessful marriage; more family conflicts; more problems with peers) [3], [5], [6], [10], [11]. Moreover, in adults with ADHD, comorbidities with other psychiatric disorders - particularly with mood disorders, anxiety disorders, antisocial personality disorder and substance use disorder [2], [6], [8], [9], [12] - are common and can lead to further impairment.

The functional impairment and poor mental health that many experience can have a cumulative effect over the course of one’s life [13], even in those who only exhibit a few ADHD symptoms and thereby fail to meet enough criteria for a clinical diagnosis [8], [14]. Thus, achieving better insights into the prevalence of ADHD and its associated factors in young adults may be crucial to preventing negative consequences and reducing illness burdens during later phases of life.

To date, the prevalence of ADHD has mainly been investigated in children and adolescents. In the limited number of studies that have focused on adults, prevalence estimates for ADHD have varied considerably, ranging from 1.0 to 7.5% (For a review, see [15], [16]), though even higher rates have been uncovered when more liberal diagnostic criteria were applied [4], [14], [16], [17]. Most of these studies were potentially biased, however, due to their use of convenience samples, drawing from particular communities, families, or from student or patient populations [3], [14], [16]–[20] rather than enrolling samples more representative of the community [16]. Only a few studies have assessed prevalence within more representative samples [6], [8]–[10], [21], [22]. However, some of these studies [6], [10], [21] utilized indirect methods (e.g., multiple imputation) by which prevalence was estimated only in a small subset of participants, and the attained estimates then extrapolated to the entire sample [16].

To date, no published epidemiological data on adult ADHD in Switzerland exist. In this country, prevalence estimates have been limited to children within the age range of 7 to 17 years. In that group, the prevalence was 5.2% (6.1% boys, 3.3% girls), meaning that it is one of the most common psychiatric disorders observed in this age group [23].

The aims of the present study were (1) to measure the prevalence of adult ADHD within a large, representative sample of young Swiss men; and (2) to identify factors associated with this disorder in this age-group. To our knowledge, this is the largest sample of young men used in a population-based study in Europe to estimate the prevalence of adult ADHD.

## Materials and Methods

### Study Design

The present study used data drawn from the *‘Cohort Study on Substance Use Risk Factors’ (C-SURF*), a longitudinal study designed to assess substance use patterns within a cohort of young Swiss men. The study was approved by the Ethics Committee for Clinical Research at Lausanne University Medical School (protocol number 15/07) and informed written consent was obtained from the participants.

The sample was recruited between August 2010 and November 2011 at three of a total of six centres that recruit men for military service, covering 21 of 26 Swiss cantons (including all French-speaking cantons). Switzerland has a mandatory army recruitment process, such that all Swiss men must submit to a formal evaluation to determine their eligibility for military service, civil service, or no service at approximately age 19. Both those who were deemed eligible to serve in the army and those deemed ineligible for military service were eligible for enrolment in our study. As there is no pre-selection to army conscription, a representative sample of young Swiss men was thereby provided for the study. It is important to note that the army centres were used only to enrol participants into the study. Both the study itself and the men’s decision to participate were entirely independent of the army. The present study used data collected during the baseline assessment only.

### Participants

A total of 7,563 conscripts gave informed consent to participate in the study. Among them, 5,990 (79.2%) completed the baseline questionnaire. A further 334 conscripts were excluded from the analysis due to missing data, so that the final sample consisted of 5,656 (74.8%) subjects.

### Assessment of ADHD

Adult ADHD was assessed using the Adult ADHD Self-Report Scale Screener (ASRS-v1.1), developed by the World Health Organization (WHO) [24], [25]. This instrument includes six questions about ADHD symptoms, which are based upon DSM-IV diagnostic criteria for ADHD. For this study, all questions referred to the last 12 months. Each item was rated on a five-point scale, from “never” (0) to “very often” (4), with responses summed up to generate a summary score ranging from 0 to 24. The answers then were dichotomized into the variable “no ADHD” (scores 0–13) and “ADHD” (scores 14–24). In order to distinguish those with borderline scores from those with more extreme scores, a four-strata classification system [25] also was utilized, with subjects assigned to the four strata as follows: 0–9 “stratum I”, 10–13 “stratum II”, 14–17 “stratum III”, and 18–24 “stratum IV”.

Participants who failed to answer at least three questions from the ASRS Screener were excluded (n = 20, 0.4%). In contrast, when participants failed to answer only one or two questions, these missing responses were replaced by means of nearest-neighbour hot-deck imputation, using a random recursive partitioning (RRP) dissimilarity matrix. This method was implemented within the RRP package [26] running in version 2.15 of the R statistical environment (R Team Core Development, 17).

### Assessment of Socio-demographic Variables and Childhood Factors

Socio-demographic variables included age (‘younger than 20 years’ vs. ‘20 years or older’), linguistic region (‘German-’ vs. ‘French-speaking’), residence (‘rural’ vs. ‘urban’), marital status (‘in relationship’ vs. ‘single’), highest achieved education (‘primary school’ vs. ‘secondary vocational school’ vs. ‘high school/university’), and degree of financial autonomy (‘financial autonomy’ vs. ‘partial financial dependency’ vs. ‘financial dependency’). Additionally, several socio-demographic variables related to the subject’s childhood were assessed, including maternal education (‘primary school’ vs. ‘secondary vocational school’ vs. ‘high school/university’), family affluence (‘above average’ vs. ‘average’ vs. ‘below average’), living arrangement before the age of 18 (‘living with biological parents’ vs. ‘living with others’), and separation of parents before 18 years of age (‘no’ vs. ‘yes’).

Furthermore, family histories for alcohol abuse and psychiatric problems were assessed for the mother, father and siblings, as well as for paternal and maternal grandparents, uncles and aunts, based upon questions adopted from the Addiction Severity Index (ASI) [27]. Answers for family members on the mother’s side were combined to generate two dichotomous variables, one for alcohol (‘yes’ vs. ‘no’) and one for psychiatric problems (‘yes’ vs. ‘no’). The same procedure was applied to family members on the father’s side and siblings. In order to reduce the number of predictors for logistic regression, answers for all family members were combined to generate two dichotomous variables for the whole family, one for alcohol (‘yes’ vs. ‘no’) and one for psychiatric problems (‘yes’ vs. ‘no’).

### Assessment of Co-morbidity

#### Major depression

Major Depression (MD) was assessed using the Major Depressive Inventory (ICD-10) – WHO-MDI [28], [29], which is a 10-item screening instrument that uses a 6-point scale for responses that range from “never” (1) to “all the time” (6). MDI items were first dichotomised to indicate the absence (0) or presence (1) of each symptom, and afterwards coded according to the DSM-IV criteria to generate a binary variable: “no MD” vs. “MD”. In accordance with the DSM-IV, MD was defined as the presence of at least five MDI items, with either item 1 or item 2 required to be among those five items [28]. Scores were computed when at least 8 of the 10 questions were answered.

#### Anti-Social Personality Disorder (ASPD)

Symptomatology of anti-social personality disorder was measured via the Mini International Neuropsychiatric Interview (MINI plus, [30]). Responses were recorded using a 6-point scale, ranging from “never” (1) to “20 times or more” (6). Afterwards, answers were dichotomised to indicate the absence (0) or presence (1) of each symptom and further coded to generate a binary variable: “no ASPD” vs. “ASPD”. In accordance with the MINI plus, ASPD was defined as the presence of two symptoms, both before and after the age of 15. Cases were excluded when more than half of the questions were not answered for before and/or after the age of 15.

#### Alcohol abuse and dependence

Twelve-month diagnoses of alcohol abuse and dependence were assessed by questionnaires [31] based on DSM-IV diagnostic criteria. The questions were originally adapted from the Semi-Structured Assessment for the Genetics of Alcoholism (SSAGA) [32], [33]. The presence of alcohol abuse was defined as a positive response to any one of the four abuse criteria and the absence of a dependence diagnosis. An alcohol dependence diagnosis was defined as a positive response to any three or more of seven dependence criteria [31].

### Statistical Analysis

All statistical analyses were performed using the statistical package SPSS 20.0. We used contingency tables to present 12-month prevalence rates for adult ADHD, as well as for socio-demographic characteristics, childhood factors, and comorbidities. Variables relating to socio-demographic characteristics, family history, and co-morbidity were compared between participants with and without ADHD using Pearson chi-square analysis. Logistic regression analyses were conducted to examine the association between adult ADHD (dependent variable) and socio-demographic variables, including family history for the whole family. Unadjusted and adjusted odds ratios (OR) and 95% confidence intervals (95% CI) were calculated for all predictors. Adjusted values were assessed using all socio-demographic variables and family histories of alcohol and psychiatric problems for the whole family as covariates.

## Results

### Prevalence of ADHD

The socio-demographic characteristics of the study sample are summarized in Table 1 (columns 1 and 2).

**Table 1:** Description of the study sample and correlates of adult ADHD; logistic regression analyses with ADHD as the outcome variable.

	Participants withcharacteristic	Prevalence ofADHD withincharacteristic	Unadjusted	Adjusted^a^
	% (n = 5656)	% (n = 226)	Odds ratio [95% CI]	Odds ratio [95% CI]
**Age** b				
<20	60.0	3.4	1.00	
≥20	40.0	4.9	1.45 [1.11, 1.89]*	1.35 [1.02, 1.80]*
**Linguistic region**				
German	45.2	2.7	1.00	
French	54.8	5.1	1.96 [1.47, 2.62]**	1.75 [1.27, 2.40]**
**Residence**				
rural	33.0	3.4	1.00	
urban	67.0	4.3	1.26 [0.94, 1.69]	1.00 [0.74, 1.36]
**Marital status** c				
in relationship	5.1	2.8		
single	94.9	4.1		
**Education**				
primary school	50.0	3.9	1.00	
secondary vocational school	28.5	3.2	0.82 [0.58, 1.14]	0.80 [0.56, 1.15]
high school/university	21.5	5.2	1.34 [0.98, 1.84]†	0.97 [0.69, 1.37]
**Financial autonomy**				
financial autonomy	23.5	2.7	1.00	
partial financial dependency	42.7	4.0	1.49 [1.01, 2.20]*	1.36 [0.91, 2.04]
financial dependency	33.8	4.9	1.86 [1.26, 2.75]*	1.78 [1.16, 2.72]*
**Living arrangement** d				
biological parents	78.1	3.8	1.00	
others	21.9	4.7	1.24 [0.91, 1.68]	1.02 [0.74, 1.41]
**Separation of parents** c **^,^** d				
no	74.5	3.8	1.00	
before birth	1.5	5.8	1.55 [0.62, 3.89]	
after birth	24.0	4.4	1.16 [0.86, 1.58]	
**Mother’s education**				
primary school	13.5	5.4	1.00	
secondary vocational school	62.2	3.2	0.59 [0.41, 0.84]*	0.64 [0.44, 0.94]*
high school/university	24.3	5.2	0.98 [0.66, 1.45]	0.94 [0.61, 1.43]
**Family affluence**				
above average	44.5	3.7	1.00	
average	41.1	4.0	1.09 [0.81, 1.46]	1.06 [0.78, 1.45]
below average	14.4	4.7	1.26 [0.86, 1.85]	1.13 [0.75, 1.72]
**Family history**				
**Problems with alcohol**				
no	76.2	3.4	1.00	
yes	23.8	6.0	1.84 [1.39, 2.44]**	1.46 [1.09, 1.96]*
**Psychiatric disease**				
no	85.0	3.4	1.00	
yes	15.0	7.6	2.39 [1.77, 3.21]**	2.13 [1.56, 2.92]**

† p<.10,

*p<.05,

**p<.001;

a adjusted for all variables;

b age range: 17–28;

c no logistic regression analyses were performed due to the small number of participants in one of the variable’s categories;

d before 18 years of age.

The 12-month prevalence of ADHD was 4.0% (n = 226). Using the four-strata classification system, no ADHD was reported in 81.3% of participants, while 14.7%, 3.4%, 0.6% of the participants were classified within strata II, III and IV, respectively, representing possible through definite cases of ADHD (Table 2).

**Table 2:** 12-month prevalence of adult ADHD in young Swiss men.

	Prevalence	
ASRS CLASSIFICATION	n	%
**Dichotomized Classification**		
ADHD (score = 14–24)	226	4.0
no ADHD (score = 0–13)	5430	96.0
**4-Strata Classification**		
I (score = 0–9)	4599	81.3
II (score = 10–13)	831	14.7
III (score = 14–17)	192	3.4
IV (score = 18–24)	34	0.6

More than half (50.8 to 65.3%) of the sample reported at least one ADHD symptom “rarely”, “sometimes”, “often” or “very often”. Symptoms of hyperactivity were reported slightly more frequently than the majority of inattention symptoms (56.0 to 65.3% and 50.8 to 61.4%, respectively). Symptoms of inattention were reported as occurring “often” or “very often” in 0.7 to 9.0% men and hyperactivity symptoms in 1.9 to 13.6%. The frequencies of each symptom are shown in Table 3.

**Table 3:** Frequency of ADHD symptoms, as assessed with the ASRS Screener.

	Total		Often		Very often	
	N	%	n	%	n	%
**Inattention symptoms**						
How often do you have trouble wrapping up the fine details of a project,once the challenging parts have been done?	3068	54.2	216	3.8	41	0.7
How often do have difficulty getting things in order when you have to do atask that requires organization?	2875	50.8	216	3.8	41	0.7
How often do you have problems remembering appointments or obligations?	2880	50.9	201	3.6	46	0.8
When you have a task that requires a lot of thought, how often do youavoid or delay getting started?	3466	61.4	507	9.0	134	2.4
**Hyperactivity symptoms**						
How often do you fidget or squirm with your hands or your feet when you haveto sit down for a long time?	3696	65.3	769	13.6	359	6.3
How often do you feel overly active and compelled to do things, like youwere driven by a motor?	3169	56.0	383	6.8	106	1.9

Total: Total number of men experiencing symptoms “rarely”, “sometimes”, “often” or “very often”.

### Socio-demographic Variables and Childhood Factors

A positive association with ADHD was found for subject age, linguistic region, education level, and degree of financial autonomy. Older subjects and those from French-speaking regions were more likely to report ADHD symptoms than those who were younger or from German-speaking regions. For education, a trend (p<0.10) was observed, with a high school/university level education associated with a higher prevalence of ADHD than having completed primary school only; however, this association failed to achieve statistical significance after adjusting for all other variables. Additionally, compared to financial autonomy, ADHD was positively related to partial or complete financial dependency. However, when adjusting for all other variables, only complete financial dependency was significant. No significant difference between participants with and without ADHD was identified for residence or marital status.

For childhood factors, logistic regression analysis revealed that subjects whose mothers had completed high school/university or just primary school were more likely to report ADHD than those whose mothers completed secondary school. Frequencies for these two former education levels were similar. Reporting a family history of alcohol or psychiatric problems was significantly associated with a higher prevalence of ADHD than not having such a history. Family affluence, living arrangement before the age of 18, and separation of parents before 18 years of age did not differ between those with and without ADHD. Table 1 presents unadjusted and adjusted ORs and 95% CI for correlates of adult ADHD.

### Co-morbid Conditions

**Figure 1:**
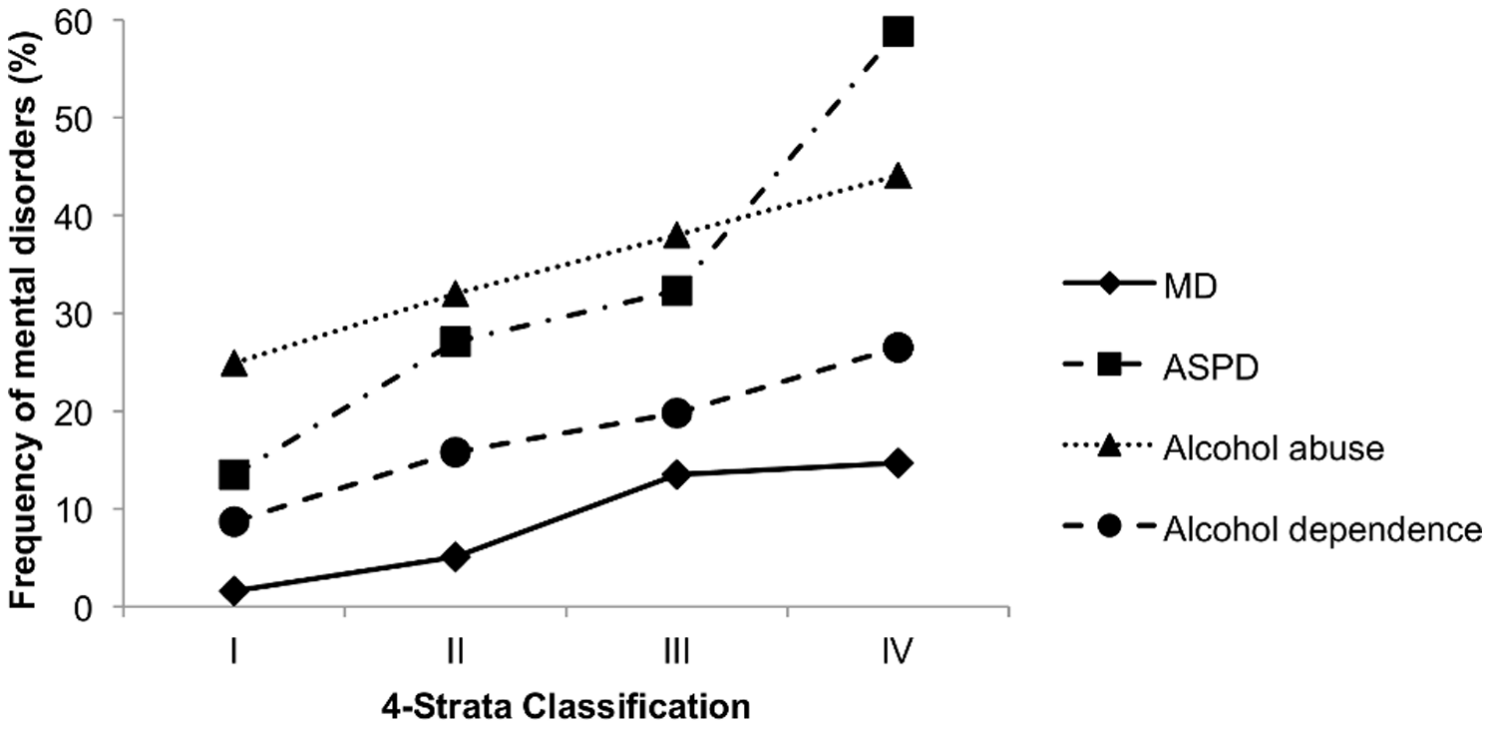
Relationship between ASRS strata and the presence of MD, ASPD, alcohol abuse and dependence.

Using the dichotomous classification (“no ADHD” vs. “ADHD”) 13.7% of our subjects with ADHD reported MD, 36.3% ASPD, 38.9% alcohol abuse, and 20.8% alcohol dependence. Rates for all these disorders were significantly higher among those with than those without ADHD (MD: X^2^ = 114.94, p<.001; ASPD: X^2^ = 67.99, p<.001; alcohol abuse and dependence X^2^ = 58.90, p<.001). Similarly, a higher percentage of men in strata II, III and IV reported the presence of MD, ASPD, alcohol abuse and dependence than men allocated to stratum I. The rate of having at least one of these psychiatric disorders systematically increased from strata I to IV (Figure 1), indicating that men with more ADHD symptoms are more likely to suffer from other psychiatric disorders as well. Rates for these co-morbid disorders, per stratum, are presented in Table 4.

**Table 4:** Relationship between ADHD and the presence of MD, ASPD, alcohol abuse and dependence.

	I	II	III	IV	With ADHD
**MD**	1.6	5.1	13.5	14.7	13.7
**ASPD**	13.5	27.1	32.3	58.8	36.3
**Alcohol abuse**	24.9	32.0	38.0	44.1	38.9
**Alcohol dependence**	8.7	15.8	19.8	26.5	20.8

## Discussion

### Prevalence of ADHD

The present study aimed to assess the prevalence of adult ADHD in a large, representative sample of young men living in Switzerland, and to examine factors associated with ADHD. In our sample, the prevalence of adult ADHD was 4.0%. This rate is midway between the rates of 1.0 to 7.5% reported in previous studies [15], [16]. Although we assessed prevalence only by applying a self-report screening questionnaire, our rate was similar to the average rate of 4.2% that was reported for higher income countries in a WHO-orchestrated international survey [6]. In the WHO study, more accurate diagnostic assessments were performed at least for a small portion of the recruited US sample, and multiple imputations were applied to estimate prevalence across all other participating countries. Our results are also in line with the crude prevalence estimate of 4.7% reported recently for a representative sample of the German population [9], in a study in which a variety of self-report instruments were used. Relative to the only pre-existent prevalence estimates for Switzerland, specifically for youths between the ages of 7 and 17 (6.2%) [23], our rates are somewhat lower. However, the previous estimate only incorporated the city of Zurich.

### Socio-demographic Variables

Contradicting earlier findings [6], [9], [16], [20], the prevalence of ADHD increased with age in our sample. By analysing the rates of adult ADHD for each age separately, we noticed that they fluctuated from age to age among older subjects (≥20 years) (data not shown). For example, a higher rate of adult ADHD was found among those who were 20 years old (5.4%) than those who were 21 (3.7%). The latter rate was similar to the rates obtained for subjects aged 18 (3.1%) and 19 (3.8), who were assigned to the younger of our two dichotomized age groups (<20 years). One possible explanation for this result may be our use of self-reports to diagnose ADHD, as self-reports may be less reliable than informant/proxy reports (e.g. parents, teachers) because people with ADHD exhibit diminished self-appraisal/awareness and thereby tend to underreport their own symptoms [34], [35]. Nevertheless, it is also possible that critical life periods may lead people with ADHD to increasingly having to confront their difficulties and, hence, be more likely to self-report symptoms. For instance, at the age of 20 years, most Swiss people start university or their first job after training, which may be experienced as rather challenging, potentially contributing to the higher prevalence rates that we observed.

We noted no significant differences in the prevalence of ADHD between those with a rural versus urban residence. This result is consistent with some previous findings [10], but stands in contrast with other prior studies identifying higher rates in urban [4] or rural areas [9]. This inconsistency might be due to differences in how rural and urban were defined in the different studies, as well as to differences in the nature of the rural and urban areas themselves [36].

Linguistic region was associated with adult ADHD, in that a higher prevalence was discovered among French-speaking versus German-speaking conscripts. Differences in prevalence between countries with different cultures have already been reported [6], [18], as well as between culturally-diverse populations within the same country [37]. These differences could be explained by differences in knowledge, understanding, and the assessment and management of ADHD symptoms between different countries and cultures. For example, a given symptom might be considered deviant in some cultures, while more accepted in others. This could affect the reports of informants [18], [37], [38]. Since how ADHD is understood, diagnosed and managed is similar throughout Switzerland, at least among health professionals and in schools, we suspect that cultural differences in response behaviours were primarily responsible for the linguistic effect we observed. French-speaking conscripts, for instance, seem to give more extreme responses than those who speak German, a pattern that was observed for every ADHD item (data not shown) as well as for other variables [39].

For our analysis, highest achieved education and financial autonomy were used as markers of professional achievement. Men with adult ADHD were more likely to have completed high school or attended university (p<0.10), although this association failed to achieve statistical significance after adjusting for all other variables. Our results are clearly contrary to most previous reports, which constantly revealed a positive association between ADHD and lower education level [3], [6], [7], [9]. It is possible that enhanced identification and referral of cases with ADHD in Switzerland, as well as more accessible and effective treatment during childhood and adolescence, might have allowed affected individuals to achieve higher levels of education than in other countries. However, it must be emphasized that, consistent with the findings of others [7], in our sample adult ADHD was positively related to partial and, even more so, to complete financial dependency. The observed positive relationship between adult ADHD and financial dependency may suggest that adults with ADHD still are impaired in some aspects of their professional life, despite having achieved a similar education level as those without ADHD. Thus, they may need more support to manage tasks at work.

In contrast to previous studies, in which problems with personal relationships were reported [3], [6], [10], in our sample no significant difference was found in marital status between those with and without ADHD. This failure to identify any significant marital difficulties could be due to the definition of marital status we used: being in a relationship was defined as being married or living with a partner, and not just having a girlfriend. Due to the young age of our participants, only a small number of subjects fulfilled our requirement for ‘marriage’ (286/5656), meaning that the number of partnered subjects with ADHD (8/226) was too small to allow for meaningful analysis.

### Childhood Factors

We did find that both a subject’s mother’s level of education and a family history of alcohol or psychiatric problems differed between those with and without adult ADHD. Mothers of participants with adult ADHD were more likely to have either a primary school or high school/university level education, while those without ADHD more often had mothers who had completed secondary school but not beyond. The finding that a low level of maternal education was associated with a greater prevalence of adult ADHD is consistent with the results of other studies, in which a higher risk for ADHD in children was observed in those with less-educated parents [40], [41]. In this context, it has been claimed that, due to its strong genetic component, parents of affected children may be more likely to have had ADHD themselves and, as a consequence, to have not been able to achieve higher education levels [41]. In contrast, the observation that a higher level of maternal education was associated with a greater prevalence of ADHD in offspring has not been reported before; but it is consistent with what Gau et al. [42] have found: a positive association between the number of psychiatric referrals and higher maternal education levels in children with ADHD. This, in turn, suggests that mothers of higher education may be more likely to be aware of their children’s problematic behaviours and have more knowledge about ADHD. One could speculate that higher parental awareness of ADHD might help their offspring to better identify and report their own symptoms.

Family history of either alcohol or psychiatric problems also was positively associated with adult ADHD. In fact, a family history of psychiatric problems exhibited the highest odds ratio for ADHD of any variable, on logistic regression analysis. Once again, the strong genetic component of ADHD and the high comorbidity of ADHD with alcohol use disorders and other mental disorders could explain this association [2]. Additionally, the difficulties and stress inherent to raising a child with ADHD could affect family functioning and the well-being of family members (for a Review, see [43]). For instance, ADHD in adolescents is linked to more depressive symptoms in mothers [44] and to more alcohol use disorders in parents [44]–[46]. These findings are even stronger when parents of affected children have ADHD themselves [44], [45]. Thus, a higher rate of family alcohol abuse and psychiatric problems may be induced by both genetic factors and challenges in the interactions of family members with the ADHD-affected individual. As such, further efforts should be undertaken to include all family members in the treatment of ADHD, and to provide special interventions for families with more than one ADHD-affected individual.

### Co-morbid Conditions

Consistent with previously-published findings [2], [6], [8]–[10], [12], a considerable proportion of our men with ADHD appeared to suffer from major depression, anti-social personality disorder, and either alcohol abuse or dependence. Additionally, men with more ADHD symptoms appear to be more likely to suffer from one of these psychiatric disorders [8]. In our sample, among those with the highest number of ADHD symptoms, the probability of having MD was nine times greater than among those in the lowest strata, in which any ADHD cases are expected. For ASPD, alcohol abuse and dependence, the rates were approximately four, two, and three times greater in the highest vs. lowest stratum, respectively. For all these psychiatric disorders, an elevated rate also was identified in those with few ADHD symptoms [8], [14]. Our results, combined with previous findings, highlight the considerable impairments in mental health that young men with ADHD can experience, as well as the need for health care providers to offer appropriate interventions for this population to reduce further illness burdens.

### Study Limitations

The following limitations must be considered relating to the currently-presented study. First, women were not included in our sample. Although some study results suggest that men and women experience ADHD in a similar way [8], inconsistent results in ADHD prevalence estimates have been reported comparing adult males and females. The reasons behind these inconsistent results remain unclear and must be clarified in future studies.

Second, the prevalence of ADHD was assessed in the context of a population-based study that was not initially intended or designed to address ADHD-related questions. Therefore, prevalence only was assessed by means of self-report using the ASRS Screener and did not further include any confirmatory diagnostic assessment. This also was true for the assessment of MD, ASPD, alcohol abuse and alcohol dependence, as well as for family histories of alcohol and psychiatric problems. However, the assessment instruments we used are all well validated. Thus, we feel that our study provides a reasonable approximation of the prevalence of ADHD among young Swiss men. Nevertheless, it is important to note that, despite excellent concordance between the ASRS Screener and a clinical diagnosis of ADHD [24], [25], the ASRS is based upon the DSM-IV criteria originally developed for children and might not provide appropriate information for the diagnosis of adult ADHD. Indeed, some study results suggest that these criteria are too conservative for adults [4], [20]. As a consequence, the true prevalence of ADHD might be slightly underestimated in our study.

Third, the medical history for ADHD, mental disorders and family histories of alcohol and psychiatric problems, was not assessed; consequently, we do not know the clinical status of our study participants or whether they had received or continued to receive any treatment.

Finally, since our data were collected at a single time point and not longitudinally, we cannot draw causal inferences.

In summary, we found that roughly 4.0% of young Swiss men have ADHD, and that associations exist with other mental disorders, like major depression and alcohol abuse, as well as with family histories of alcohol and psychiatric problems. ADHD adversely affects work, health and interpersonal relationships. As such, clinicians and school professionals should remain alert to its existence and consequences in young males.

## References

[1] American Psychiatric Association (1994) Diagnostic and statistical manual of mental disorders (DSM-IV). 4th rev. ed. American Psychiatric Press, Washington, D.C.

[2] Faraone SV, Biederman J, Spencer T, Wilens T, Seidman LJ, et al (2000) Attention-deficit/hyperactivity disorder in adults: an overview. Biol Psychiatry 48: 9–20. 10913503 10.1016/s0006-3223(00)00889-1

[3] Ebejer JL, Medland SE, van der Werf J, Gondro C, Henders AK, et al (2012) Attention deficit hyperactivity disorder in Australian adults: prevalence, persistence, conduct problems and disadvantage. PloS One 7: e47404. 23071800 10.1371/journal.pone.0047404 PMC3468512

[4] Faraone SV, Biederman J (2005) What is the prevalence of adult ADHD? Results of a population screen of 966 adults. J Atten Disord 9: 384–391. 16371661 10.1177/1087054705281478

[5] Biederman J, Faraone S (2006) The effects of attention-deficit/hyperactivity disorder on employment and household income. MedGenMed 8: 12. PMC1781280 17406154

[6] Fayyad J, De Graaf R, Kessler R, Alonso J, Angermeyer M, et al (2007) Cross-national prevalence and correlates of adult attention-deficit hyperactivity disorder. Br J Psychiatry 190: 402–409. 17470954 10.1192/bjp.bp.106.034389

[7] Biederman J, Petty CR, Woodworth KY, Lomedico A, Hyder LL, et al (2012) Adult outcome of attention-deficit/hyperactivity disorder: A controlled 16-year follow-up study. J Clin Psychiat 73: 941–950. 10.4088/JCP.11m07529 22901345

[8] Das D, Cherbuin N, Butterworth P, Anstey KJ, Easteal S (2012) A population-based study of attention deficit/hyperactivity disorder symptoms and associated impairment in middle-aged adults. PloS One 7: e31500. 22347487 10.1371/journal.pone.0031500 PMC3275565

[9] de Zwaan M, Gruss B, Muller A, Graap H, Martin A, et al (2012) The estimated prevalence and correlates of adult ADHD in a German community sample. Eur Arch Psychiatry Clin Neurosci 262: 79–86. 21499942 10.1007/s00406-011-0211-9

[10] Kessler RC, Adler L, Barkley R, Biederman J, Conners CK, et al (2006) The prevalence and correlates of adult ADHD in the United States: results from the National Comorbidity Survey Replication. Am J Psychiatry 163: 716–723. 16585449 10.1176/appi.ajp.163.4.716 PMC2859678

[11] Rosler M, Casas M, Konofal E, Buitelaar J (2010) Attention deficit hyperactivity disorder in adults. World J Biol Psychiatry 11: 684–698. 20521876 10.3109/15622975.2010.483249

[12] Moss SB, Nair R, Vallarino A, Wang S (2007) Attention deficit/hyperactivity disorder in adults. Prim Care Clin Office Pract 34: 445–473. 10.1016/j.pop.2007.05.005 17868755

[13] Brod M, Schmitt E, Goodwin M, Hodgkins P, Niebler G (2012) ADHD burden of illness in older adults: a life course perspective. Qual Life Res 21: 795–799. 21805205 10.1007/s11136-011-9981-9

[14] Kooij JJ, Buitelaar JK, van den Oord EJ, Furer JW, Rijnders CA, et al (2005) Internal and external validity of attention-deficit hyperactivity disorder in a population-based sample of adults. Psychol Med 35: 817–827. 15997602 10.1017/s003329170400337x

[15] Willcutt EG (2012) The prevalence of DSM-IV attention-deficit/hyperactivity disorder: a meta-analytic review. Neurotherapeutics 9: 490–499. 22976615 10.1007/s13311-012-0135-8 PMC3441936

[16] Simon V, Czobor P, Balint S, Meszaros A, Bitter I (2009) Prevalence and correlates of adult attention-deficit hyperactivity disorder: meta-analysis. British J Psychiatry 194: 204–211. 10.1192/bjp.bp.107.048827 19252145

[17] Heiligenstein E, Conyers LM, Berns AR, Miller MA (1998) Preliminary normative data on DSM-IV attention deficit hyperactivity disorder in college students. J Am Coll Health 46: 185–188. 9519582 10.1080/07448489809595609

[18] DuPaul GJ, Schaughency EA, Weyandt LL, Tripp G, Kiesner J, et al (2001) Self-report of ADHD symptoms in university students: cross-gender and cross-national prevalence. J Learn Disabil 34: 370–379. 15503581 10.1177/002221940103400412

[19] Almeida Montes LG, Hernandez Garcia AO, Ricardo-Garcell J (2007) ADHD prevalence in adult outpatients with nonpsychotic psychiatric illnesses. J Atten Disord 11: 150–156. 17709815 10.1177/1087054707304428

[20] Bitter I, Simon V, Balint S, Meszaros A, Czobor P (2010) How do different diagnostic criteria, age and gender affect the prevalence of attention deficit hyperactivity disorder in adults? An epidemiological study in a Hungarian community sample. Eur Arch Psychiatry Clin Neurosci 260: 287–296. 19806424 10.1007/s00406-009-0076-3

[21] Medina-Mora ME, Borges G, Lara C, Benjet C, Blanco J, et al (2005) Prevalence, service use, and demographic correlates of 12-month DSM-IV psychiatric disorders in Mexico: results from the Mexican National Comorbidity Survey. Psychol Med 35: 1773–1783. 16300691 10.1017/S0033291705005672

[22] Bernardi S, Faraone SV, Cortese S, Kerridge BT, Pallanti S, et al (2012) The lifetime impact of attention deficit hyperactivity disorder: results from the National Epidemiologic Survey on Alcohol and Related Conditions (NESARC). Psychol Med 42: 875–887. 21846424 10.1017/S003329171100153X PMC3383088

[23] Steinhausen HC, Winkler C, Meier MM, Kannenberg R (1998) Prevalence of child and adolescent psychiatric disorders: the Zurich epidemiological study. Acta Psychiatr Scand 98: 262–271. 9821446 10.1111/j.1600-0447.1998.tb10082.x

[24] Kessler RC, Adler L, Ames M, Demler O, Faraone S, et al (2005) The World Health Organization Adult ADHD Self-Report Scale (ASRS): a short screening scale for use in the general population. Psychol Med 35: 245–256. 15841682 10.1017/s0033291704002892

[25] Kessler RC, Adler LA, Gruber MJ, Sarawate CA, Spencer T, et al (2007) Validity of the World Health Organization Adult ADHD Self-Report Scale (ASRS) Screener in a representative sample of health plan members. Int J Methods Psychiatr Res 16: 52–65. 17623385 10.1002/mpr.208 PMC2044504

[26] Iacus SA, Porro G (2007) Missing data imputation, matching and other applications of random recursive partitioning. Computational Statistics & Data Analysis 52: 773–789.

[27] Mclealen AT, Luborsky L, Cacciola J, Griffith J, Evans F, et al (1985) New data from the Addiction Severity Index. Reliability and validity in three centers. J Nerv Ment Dis 173: 412–423. 4009158 10.1097/00005053-198507000-00005

[28] Bech P, Rasmussen NA, Olsen LR, Noerholm V, Abildgaard W (2001) The sensitivity and specificity of the Major Depression Inventory, using the Present State Examination as the index of diagnostic validity. J Affect Disord 66: 159–164. 11578668 10.1016/s0165-0327(00)00309-8

[29] Olsen LR, Jensen DV, Noerholm V, Martiny K, Bech P (2003) The internal and external validity of the Major Depression Inventory in measuring severity of depressive states. Psychol Med 33: 351–356. 12622314 10.1017/s0033291702006724

[30] Lecrubier Y, Weiller E, Hergueta T, Amorim P, Bonora LI, et al.. (1998) MINI Mini International Neuropsychiatric Interview. French version 5.00. Hopital de la Pitié Salpétriere, INSERM. Paris, France.

[31] Knight JR, Wechsler H, Kuo M, Seibring M, Weitzman ER, et al (2001) Alcohol abuse and dependence among U.S. college students. J Stud Alcohol 63: 263–270. 10.15288/jsa.2002.63.263 12086126

[32] Bucholz KK, Cadoret R, Cloninger CR, Dinwiddie SH, Hesselbrock VM, et al (1994) A new, semistructured psychiatric interview for use in genetic-linkage studies - a report on the reliability of the SSAGA. J Stud Alcohol 55: 149–158. 8189735 10.15288/jsa.1994.55.149

[33] Hesselbrock M, Easton C, Bucholz KK, Schuckit M, Hesselbrock V (1999) A validity study of the SSAGA - a comparison with the SCAN. Addiction 94: 1361–1370. 10615721 10.1046/j.1360-0443.1999.94913618.x

[34] Barkley RA, Fischer M, Smallish L, Fletcher K (2002) The persistence of attention-deficit/hyperactivity disorder into young adulthood as a function of reporting source and definition of disorder. J Abnorm Psychol 111: 279–289. 12003449

[35] Sibley MH, Pelham WE, Molina BSG, Gnagy EM, Waxmonsky JG, et al (2012) When diagnosing ADHD in young adults emphasize informant reports, DSM items, and impairment. J Consult Clin Psych 80: 1052–1061. 10.1037/a0029098 PMC3919146 22774792

[36] Peen J, Schoevers RA, Beekman AT, Dekker J (2010) The current status of urban-rural differences in psychiatric disorders. Acta Psychiatr Scand 121: 84–93. 19624573 10.1111/j.1600-0447.2009.01438.x

[37] Waite R, Ramsay JR (2010) Cultural proficiency: a Hispanic woman with ADHD - a case example. J Atten Disord 13: 424–432. 19465731 10.1177/1087054709332393

[38] Skounti M, Philalithis A, Galanakis E (2007) Variations in prevalence of attention deficit hyperactivity disorder worldwide. Eur J Pediatr 166: 117–123. 17033803 10.1007/s00431-006-0299-5

[39] Dey M, Gmel G (2013) Mohler-Kuo (2013) Body mass index and health-related quality of life among young Swiss men. BMC Public Health 13: 1028 doi:10.1186/1471-2458-13-1028 24172041 10.1186/1471-2458-13-1028 PMC3840558

[40] Ezpeleta L, de la Osa N, Domenech JM (2013) Prevalence of DSM-IV disorders, comorbidity and impairment in 3-years-old Spanish preschoolers. Soc Psychiatry Psychiatr Epidemiol doi:10.1007/s00127-013-0683-1. 10.1007/s00127-013-0683-1 23595297

[41] Cuffe SP, Moore CG, McKeown RE (2005) Prevalence and correlates of ADHD symptoms in the National Health Interview Survey. J Atten Disord 9: 392–401. 16371662 10.1177/1087054705280413

[42] Gau SSF, Lin YJ, Shang CY, Liu SK, Chiu YN, et al (2010) Emotional/behavioral problems and functional impairment in clinic- and community-based children with attention-deficit/hyperactivity disorder in Taiwan. J Abnorm Child Psychol 38: 521–532. 20069354 10.1007/s10802-009-9381-6

[43] Harpin VA (2005) The effect of ADHD on the life of an individual, their family, and community from preschool to adult life. Arch Dis Child doi:10.1136/Adc.2004.059006. 10.1136/adc.2004.059006 PMC1765272 15665153

[44] Babinski DE, Pelham WE, Molina BS, Gnagy EM, Waschbusch DA, et al.. (2012) Maternal ADHD, parenting, and psychopathology among mothers of adolescents with ADHD. J Atten Disord doi:10.1177/1087054712461688. 10.1177/1087054712461688 PMC3582708 23160485

[45] Minde K, Eakin L, Hechtman L, Ochs E, Bouffard R, et al (2003) The psychosocial functioning of children and spouses of adults with ADHD. J Child Psychol Psyc 44: 637–646. 10.1111/1469-7610.00150 12751853

[46] Pelham WE, Lang AR (1999) Can your children drive you to drink? Stress and parenting in adults interacting with children with ADHD. Alcohol Res Health 23: 292–298. 10890826 PMC6760385

